# Clonal Hematopoiesis of Indeterminate Potential From a Heart Failure Specialist's Point of View

**DOI:** 10.1161/JAHA.123.030603

**Published:** 2023-07-25

**Authors:** Maurits A. Sikking, Sophie L. V. M. Stroeks, Olivia J. Waring, Michiel T. H. M. Henkens, Niels P. Riksen, Alexander Hoischen, Stephane R. B. Heymans, Job A. J. Verdonschot

**Affiliations:** ^1^ Department of Cardiology Cardiovascular Research Institute Maastricht (CARIM), Maastricht University Medical Center (MUMC) Maastricht the Netherlands; ^2^ Department of Pathology Cardiovascular Research Institute Maastricht (CARIM), Maastricht University Medical Center (MUMC) Maastricht the Netherlands; ^3^ Netherlands Heart Institute (NLHI) Utrecht the Netherlands; ^4^ Department of Internal Medicine Radboud University Medical Center Nijmegen the Netherlands; ^5^ Department of Human Genetics Radboud University Medical Center Nijmegen the Netherlands; ^6^ Department of Cardiovascular Research University of Leuven Belgium; ^7^ Department of Clinical Genetics Maastricht University Medical Center (MUMC) Maastricht the Netherlands

**Keywords:** atherosclerotic cardiovascular disease, clonal hematopoiesis, heart failure, inflammation, Heart Failure, Cardiomyopathy, Genetics, Atherosclerosis, Inflammation

## Abstract

Clonal hematopoiesis of indeterminate potential (CHIP) is a common bone marrow abnormality induced by age‐related DNA mutations, which give rise to proinflammatory immune cells. These immune cells exacerbate atherosclerotic cardiovascular disease and may induce or accelerate heart failure. The mechanisms involved are complex but point toward a central role for proinflammatory macrophages and an inflammasome‐dependent immune response (IL‐1 [interleukin‐1] and IL‐6 [interleukin‐6]) in the atherosclerotic plaque or directly in the myocardium. Intracardiac inflammation may decrease cardiac function and induce cardiac fibrosis, even in the absence of atherosclerotic cardiovascular disease. The pathophysiology and consequences of CHIP may differ among implicated genes as well as subgroups of patients with heart failure, based on cause (ischemic versus nonischemic) and ejection fraction (reduced ejection fraction versus preserved ejection fraction). Evidence is accumulating that CHIP is associated with cardiovascular mortality in ischemic and nonischemic heart failure with reduced ejection fraction and involved in the development of heart failure with preserved ejection fraction. CHIP and corresponding inflammatory pathways provide a highly potent therapeutic target. Randomized controlled trials in patients with well‐phenotyped heart failure, where readily available anti‐inflammatory therapies are used to intervene with clonal hematopoiesis, may pave the way for a new area of heart failure treatment. The first clinical trials that target CHIP are already registered.

**Nonstandard Abbreviations and Acronyms**
CH: clonal hematopoiesis
CHDM: clonal hematopoiesis driver mutation
CHIP: clonal hematopoiesis of indeterminate potential
HFpEF: heart failure with preserved ejection fraction
HFrEF: heart failure with reduced ejection fraction
HSC: hematopoietic stem cell
NLRP3: NLR family pyrin domain containing 3
VAF: variant allele frequency

Clonal hematopoiesis (CH) refers to any clonal expansion state in the blood‐forming system. Somatic mutations may provide a selective advantage to hematopoietic stem cells (HSCs) and lead to expansion of a hematopoietic stem cell clone. In case a leukemogenic driver mutation is present in at least 4% of unnucleated blood cells (ie, excluding red blood cells and platelets), and a hematological malignancy is absent, we speak of clonal hematopoiesis of indeterminate potential (CHIP).^1^ CHIP is a common phenomenon, strongly associated with aging, and contributes to the formation of a genetically distinct subpopulation of blood cells. It occurs in hematologically healthy people and is increasingly recognized as a risk factor for a spectrum of age‐related diseases, including hematological cancers and atherosclerotic cardiovascular disease (ASCVD) (coronary heart disease and stroke).^2^, ^3^, ^4^, ^5^ Interestingly, accumulating evidence points to a role for CHIP in the development and prognosis of heart failure, in both ischemic and nonischemic causes.^6^, ^7^, ^8^, ^9^, ^10^, ^11^, ^12^, ^13^, ^14^, ^15^, ^16^, ^17^, ^18^, ^19^ Previous reviews reported on the associations of CHIP on cardiovascular disease as a whole.^20^, ^21^, ^22^, ^23^, ^24^, ^25^, ^26^, ^27^ However, most articles reporting on the association between CHIP and heart failure were reported in the past year and are not included in the previous reviews. In this review, we look at CHIP from a heart failure specialist's perspective by schematically overviewing the pathophysiology and consequences of CHIP using the left ventricle ejection fraction as a cornerstone. Furthermore, we evaluate CHIP and corresponding inflammatory pathways as treatment targets and emphasize anti‐inflammatory drugs as future therapy for patients with heart failure and CHIP.

## PATHOPHYSIOLOGY OF CHIP

Although HSCs are quiescent cells, during each cell division they are at risk to stochastically acquire coding mutations.^28^ Most of the time, these mutations are neutral; hence, they do not increase the formation of blood cells and do not increase the HSC's ability to form a clone.^29^ At other times, the mutation stimulates the HSC to either progressively expand or brings a survival advantage to the HSC or its progeny.^25^, ^29^, ^30^ Ultimately, the percentage of circulating leukocytes with these clonal hematopoiesis driver mutations (CHDMs) increases, leading to distinct subpopulations of blood cells (eg, monocytes, T cells) in the circulation.^30^, ^31^

There are currently 3 mechanisms known for mutations to cause CHIP: (1) loss of balance between self‐renewal and differentiation of HSCs,^32^, ^33^ (2) enhanced resistance of HSCs against extrinsic insults (eg, chemotherapy),^34^, ^35^, ^36^ and (3) protect against inflammation.^30^ Each CHDM may have its own mechanism that leads to HSC dominance. CHDMs are most commonly found in genes encoding epigenetic enzymes (eg, *DNMT3A*, *TET2*, and *ASXL1*), signaling proteins (eg, *JAK2*),^3^, ^4^, ^37^ spliceosome components (eg, *SRSF2* and *SF3B1*), or members of the DNA damage response (eg, *PPM1D* and *TP53*).^3^, ^4^, ^37^ Normally, differentiation signals stimulate *DNMT3A* to epigenetically turn off self‐renewal genes in HSCs and upregulate differentiation factors.^38^, ^39^ Mutations in *DNMT3A* may increase self‐renewal and lower the ability of HSCs to differentiate into progenitor cells, as was shown in mice: complete knockout of *DNMT3A* in HSCs of mice immortalized HSCs, increased self‐renewal, and reduced differentiation efficiency.^32^ Comparably, restoring *TET2* reversed aberrant self‐renewal of preleukemic HSCs.^33^ CHDMs may increase resistance against external insults. CHDMs in *PPM1D* and *p53* lead to a clonal dominance by increasing resistance to external insults (ie, only in case the external result occurs). Radiative cancer therapies, topoisomerase II inhibitors (eg, anthracyclines), or platinum therapeutics select clones with mutations in *PPM1D* and *p53*, probably by killing nonmutated HSCs, whereas these mutations provide protection for the mutated clone itself.^34^, ^35^, ^36^ Lastly, CHDMs may lead to clonal dominance by protecting against inflammation. CHDMs in *ASXL1* enhance protection of HSC offspring against inflammation while stimulating release of proinflammatory factors at the same time, thereby giving the clone an advantage against nonmutated cells.^30^

**Figure 1:**
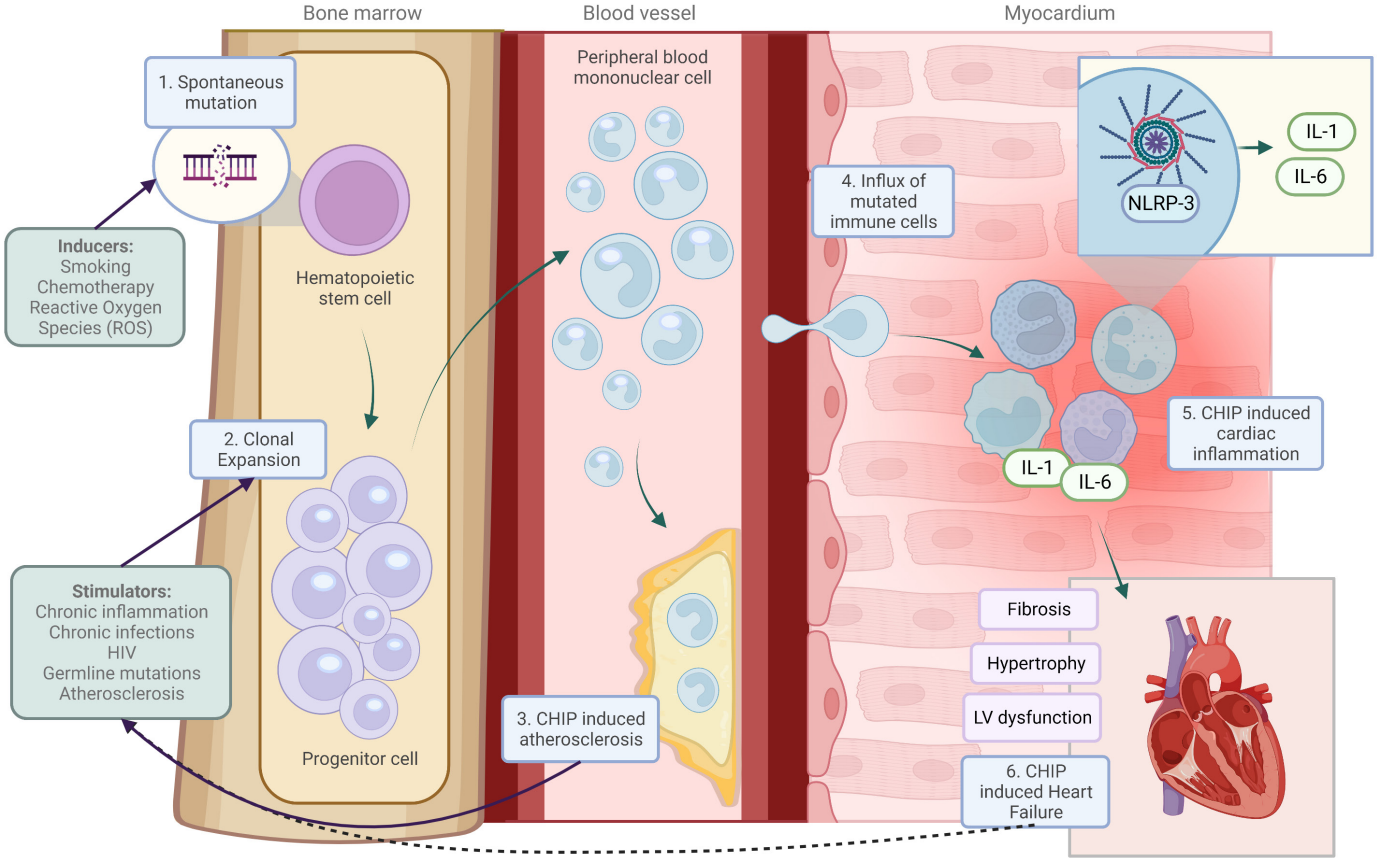
The association between clonal hematopoiesis and heart failure. Mutations in hematopoietic stem and progenitor cells give rise to clones that expand over time (1). Factors stimulate clonal proliferation (2). Consequently, these mutated cells enter the blood stream and myocardium and cause atherosclerosis (3) or impair cardiac function (4). An inflammasome/interleukin 1/6‐mediated response (5) is central in clonal hematopoiesis‐induced heart failure (6). Heart failure could be a driver of clonal proliferation, as indicated by the dashed line. Solid lines are based on published results. CHIP indicates clonal hematopoiesis of indeterminate potential; IL‐1, interleukin‐1; IL‐6, interleukin‐6; LV, left ventricular; NLRP3, NLR family pyrin domain containing 3; and ROS, reactive oxygen species.

There are specific conditions that drive clonal hematopoiesis. Although the mutation rate per DNA replication is constant,^40^ HSCs replicates while we age; hence, it is estimated that humans harbor up to 1.4 million coding mutations within the HSC pool by 70 years of age.^41^ Besides aging, other conditions that drive clonal hematopoiesis are reactive oxygen species,^42^ smoking,^43^ and chemotherapy^34^ by inducing DNA mutations, and chronic inflammation,^30^, ^44^ chronic infections,^45^ HIV,^46^ certain germline mutations,^47^, ^48^ and atherosclerosis^49^ by chronically activating HSCs to form clones (Figure 1).

Despite their varied functions, most mutated genes still associate with a comparable, proinflammatory phenotype in a wide variety of diseases. For instance, TET2 (ten‐eleven translocation 2) mediates gene transcription via DNA demethylation and indirect histone deacetylation. Loss‐of‐function *TET2* mutations lead to an increased myeloid‐led inflammatory response by 2 potential mechanisms. In the first mechanism, TET2 recruits Hdac1/2 (histone deacetylase1/2) to the *IL‐6* (interleukin‐6) promotor DNA segment.^50^ Hdac1/2 deacetylates this promotor segment, and thereby inhibits IL‐6 expression.^50^ TET2 dysfunction therefore leads to higher expression of IL‐6, especially in late‐stage inflammation, when the inflammatory trigger is already resolved.^50^ In the second mechanism, TET2 increases IL‐1 (interleukin‐1) expression either via the NLRP3 (NLR family pyrin domain containing 3)‐inflammasome or direct IL‐1 upregulation, and subsequently increases IL‐6 expression.^11^, ^14^, ^51^

CHDMs in *DNMT3A* are associated with a comparable phenotype but likely via a different intracellular mechanism, because DNMT3A regulates different genes than TET2. The exact intracellular pathways are unknown, but loss‐of‐function mutations in *DNMT3A* associated with myeloid upregulation of NLRP3 and IL‐1 and IL‐6.^16^ It is important to know that inflammation itself may stimulate dominance by *DNMT3A* mutated clones, as was shown in vivo and in vitro in mice.^52^ Transfer of *DNMT3A* mutated bone marrow cells led to a higher proportion of circulating *DNMT3A*‐mutated blood cells in aged mice (age 15 months) compared with young mice (age 2 months).^52^ The authors attribute this to age‐related inflammation and show that in vitro stimulation of HSCs by TNF‐α (tumor necrosis factor‐α) increases the proportion of *DNMT3A*‐mutated HSCs.^52^

Valine‐to‐phenylalaline mutations at amino acid 617 (V617) in the *JAK2* gene (*Jak2* ^V617F^) are gain‐of‐function mutations by upregulating of JAK2/STAT signaling. They were relatively common in a Danish population (prevalence of 3.1%) and are associated with smoking, alcohol consumption, aging, and myeloproliferative neoplasms.^53^ These mutations are associated with upregulation of AIM2 (absent in melanoma 2) inflammasome and IL‐1, at least in ASCVD.^54^ The exact mechanism by with mutations in *Jak2* ^V617f^ lead to clonal dominance is incompletely elucidated but at least drive proliferation of macrophages in the atherosclerotic plaque.^54^ A potential second mechanism by which these mutations cause disease is by increased production of neutrophil extracellular traps by *Jak2* ^V617f^‐mutated neutrophils. *Jak2* ^V617f^ mutations associate with increased risk of thrombosis potentially via neutrophil extracellular trap formation.^55^, ^56^ Inhibition of JAK–STAT signaling abrogated neutrophil extracellular trap formation and reduced thrombosis in mice carrying the *Jak2* ^V617f^ mutation.^55^

## ASSOCIATIONS BETWEEN CLONAL HEMATOPOIESIS AND ATHEROSCLEROSIS

**Figure 2:**
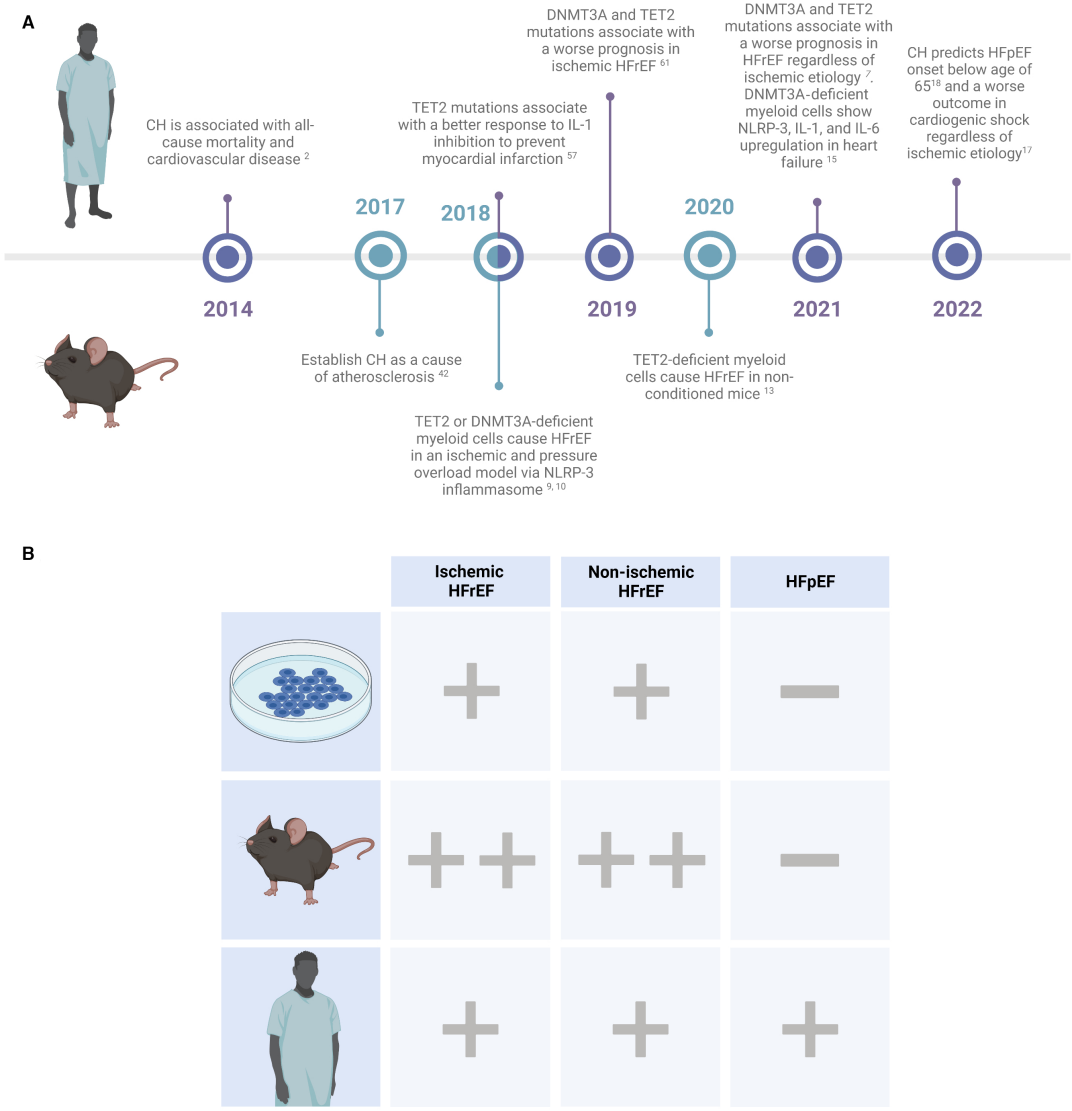
Timeline of scientific advancements in clonal hematopoiesis and overview of research currently performed across the left ventricle ejection classification. **A**, Since the first discovery of CH‐associated cardiovascular disease in 2014, research in CH grew with multiple major scientific advancements in the years thereafter. In 2019, CH was associated with a worse prognosis in patients with ischemic HFrEF. In 2020, the first mouse study that did not use any external trigger to cause heart failure (eg, pressure overload, ischemia) showed that CH by itself may lead to HFrEF. In 2021, CH was associated with a worse prognosis in patients with HFrEF regardless of ischemic cause. In 2022, CH was associated with development of HFpEF. **B**, In vitro, in vivo, and patient studies performed across nonischemic and ischemic HFrEF and HFpEF. + indicates association studies; ++, studies that established mechanisms; – the absence of research. CH indicates clonal hematopoiesis; HFpEF, heart failure with preserved ejection fraction; HFrEF, heart failure with reduced ejection fraction; IL‐1, interleukin‐1; IL‐6, interleukin‐6; and NLRP3, NLR family pyrin domain containing 3.

The detection of ischemia is one of the first steps in the diagnostic workup of new patients with heart failure. Therefore, we first outline the associations between clonal hematopoiesis and atherosclerosis. Because the association between CHIP and all‐cause mortality, coronary artery disease, and stroke was first demonstrated in 2014^3^ (Figure 2), interest in the field has surged. These associations established the role of CHIP in ASCVD.^2^, ^51^ CHIP is implicated in cardiovascular and atherosclerotic risk factors, as well as the development and progression of ischemic and nonischemic heart failure.

## INTERPLAY BETWEEN CLONAL HEMATOPOIESIS AND CARDIOVASCULAR RISK FACTORS

There is a broad range of cardiovascular risk factors associated with CHIP; however, the directionality of the relationships is difficult to determine due to their complexity. For example, it is suggested that CHIP may cause cardiometabolic complications in obesity and diabetes, but smoking may be a driver of CHIP, especially for CHDMs in *ASXL1*,^34^, ^57^ and *DNMT3A*.^57^

The effect of CHIP on the cardiometabolic complications of obesity is a complicated example of the association between CHIP and cardiovascular health. These complications are more prevalent in patients with obesity with CHIP compared with those without CHIP, even including those with diabetes,^3^, ^58^ chronic inflammation (eg, IL‐1/IL‐6),^59^ and dyslipidemia.^49^ Myeloid cells derived from mice with *Tet2* loss‐of‐function mutations are able to trigger systemic inflammation, mediated via IL‐1, in white adipose tissue. Increased insulin resistance then follows, indicating a direct effect of CH on the development of diabetes.^60^ However, circulating proinflammatory factors such as IL‐1 or IFN‐γ (interferon‐γ) have a stimulating role in clonal proliferation and expansion,^30^, ^45^, ^61^ and this systemic inflammation may stimulate CHIP through induction of clonal proliferation. Insulin resistance, in turn, may also promote the development of CHIP by stimulating clonal proliferation. A longitudinal analysis of patients with diabetes noted increased clone presence at multiple time points.^58^ Likewise, mice models showed that obesity may promote the development of CHIP by driving the growth of at least clones with mutations in *Tet2*, *Dnmt3a*, *Asxl1*, or *Jak2*.^62^

Dyslipidemia is one of the cardiometabolic sequela in obesity but may also be present in patients without obesity as part of the atherosclerosis trait complex.^49^ *Tet2*‐deficient macrophages in the atherosclerotic plaque produce more IL‐1 and IL‐6 when stimulated with low‐density lipoprotein compared with macrophages without *Tet2* mutations,^2^, ^51^ suggesting that hypercholesterolemia may increase the proinflammatory effects of CHIP. Furthermore, low‐circulating high‐density lipoprotein,^63^, ^64^ high‐intracellular cholesterol in HSCs,^49^ and atherosclerosis itself^49^, ^65^ stimulate clonal proliferation. However, clinical studies could not confirm an increased prevalence of hypercholesterolemia in patients with CHIP, suggesting that these effects are either small, not universal to all CHDMs, or independent from low‐density lipoprotein cholesterol. Furthermore, smoking is a classical cardiovascular risk factor that clearly increases the risk of CHIP.^4^, ^47^, ^57^, ^66^, ^67^, ^68^, ^69^ Smoking induces DNA mutations, and evidence suggests that it also increases hematopoietic proliferation,^70^ making it another possible cause of CHIP, especially for CHDMs in *ASXL1*.^34^, ^57^

In summary, CHIP is deeply intertwined in the development and progression of cardiovascular risk factors; however, these risk factors, in‐turn, stimulate clonal proliferation and CHIP. Cardiovascular risk management is vital, and in the future, CHIP can be taken forward for use in risk stratification and therapy.

## INTERACTION BETWEEN CLONAL HEMATOPOIESIS AND ATHEROSCLEROSIS

Studies on CHIP and atherosclerosis were the first that established its role in nonhematological diseases (Figure 2). Patients with CHIP have twice the risk for coronary artery disease and stroke,^3^ and up to 4 times the risk for early‐onset (<50 years of age) myocardial infarction,^2^ independent of cardiovascular risk factors. Even patients with already established ASCVD have a higher risk of an atherosclerotic event when they have CH.^71^ This emphasizes CHIP as an important novel risk factor for ASCVD, especially because up to 17% of patients with coronary artery disease have clonal hematopoiesis.^2^, ^72^

**Figure 3:**
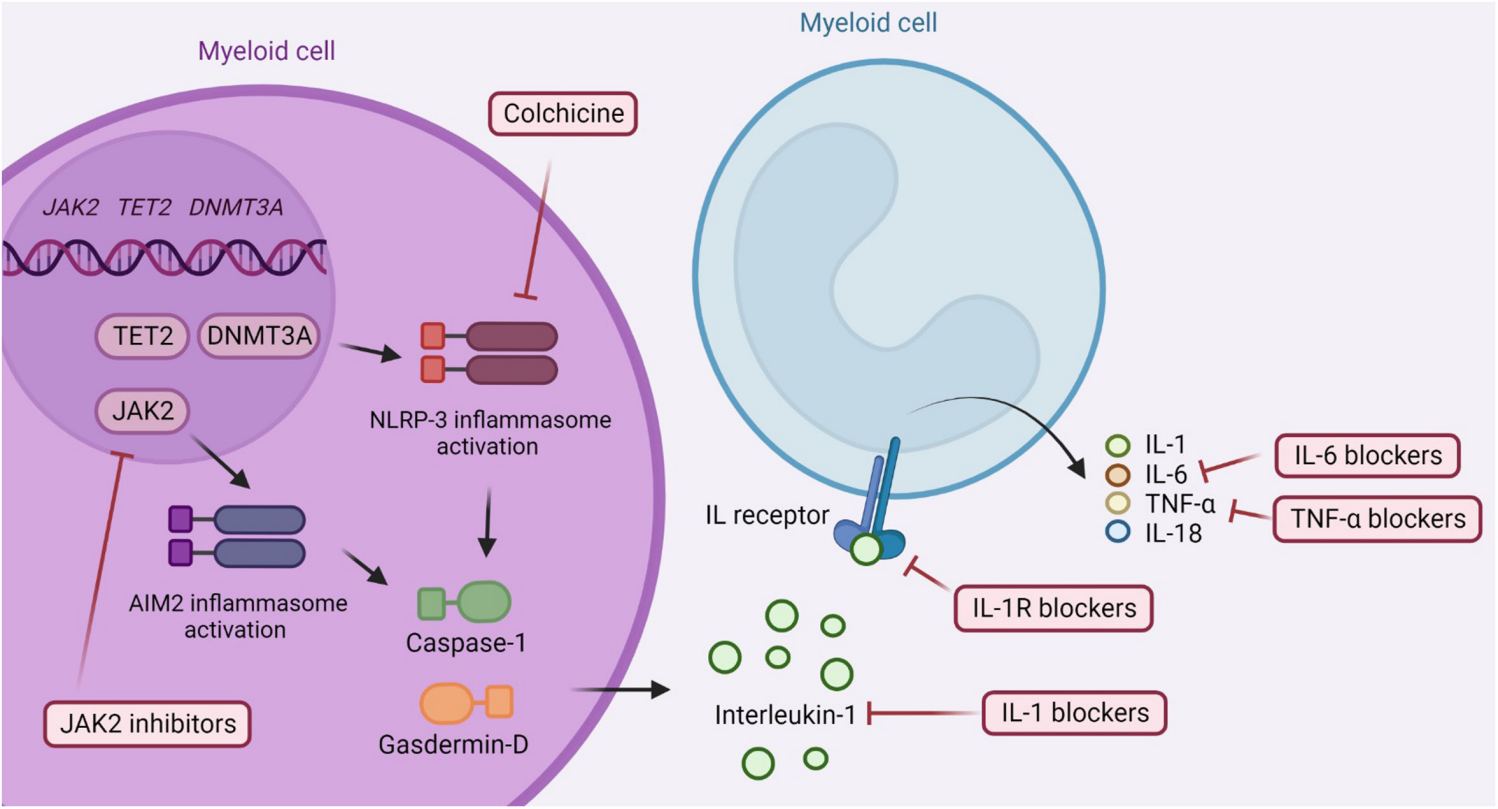
Potential treatment targets for clonal hematopoiesis‐related inflammation. Depending on the clonal hematopoiesis driver mutation involved, therapeutic targets upstream of proinflammatory cytokines may be targeted by JAK2 inhibitors (eg, in case of the gain‐of‐function mutation *JAK2* ^*V617F*^) or by NLRP3 inhibitors (eg, in case of a *TET2* or a *DNMT3A* mutation). Downstream of the inflammasome proinflammatory cytokines, such as interleukin‐1, interleukin‐6, and tumor necrosis factor, are a potential target of clonal hematopoiesis‐mediated inflammation. Purple indicates a mutated cell, and light blue indicates a normal cell. IL indicates interleukin; IL‐1, interleukin‐1; IL‐1R, interleukin‐1 receptor; IL‐6, interleukin‐6; IL‐18, interleukin‐18; NLRP3, NLR family pyrin domain containing 3; and TNF‐α, tumor necrosis factor‐α.

Three mechanisms are important in the interaction between CHIP and ASCVD: (1) CHIP upregulates the inflammasome/IL‐1/IL‐6 pathway (Figures 1 and 3). (2) CHIP increases ASCVD in a dose‐dependent manner (ie, larger mutated leukocyte clones associate with higher ASCVD risk). (3) Atherosclerosis stimulates the progression of CH.

The dependency on the inflammasome/IL‐1/IL‐6 pathway was suggested in mice,^2^, ^54^ where either *Tet2*‐deficient^2^, ^51^ or *Jak2* ^V617F^ macrophages^54^ with increased inflammasome activity^51^, ^54^ accumulated in the atherosclerotic plaque. These cells produced an inflammatory response initiated by IL‐1 and IL‐6^2^, ^51^, ^54^ production, leading to worsened plaque stability.^2^, ^51^ Importantly, inflammasome inhibitors (either an NLRP3‐inflammasome^51^ or AIM2‐inflammasome^54^ inhibitor depending on the gene involved) confirmed the involvement of the inflammasome/IL‐1/IL‐6 pathway and could restore plaque stability.^51^, ^54^ Interestingly, 2 studies went on to confirm the role of the inflammasome/IL‐1/IL‐6 pathway in humans.^44^, ^72^ Firstly, a population‐based association study used a common germline variant in the IL‐6 receptor gene (*IL6R*, p.[Asp358Ala]) as a genetic proxy of IL‐6 deficiency.^44^ CHIP was associated with a higher risk of ASCVD, but only in the absence of genetic IL‐6 signaling deficiency.^44^ This was later repeated in a larger analysis as well as in a population with ischemic stroke, showing that the variant in *IL6R* at least partially mitigates the risk of (recurrent) vascular events.^73^, ^74^ Secondly, there are already promising data from Canakinumab Anti‐inflammatory Thrombosis Outcomes Study on the use of canakinumab (a monoclonal antibody directed at IL‐1β) for prevention of major adverse cardiovascular events in patients with previous myocardial infarction and increased C‐reactive protein.^72^, ^75^ In an exploratory secondary analysis, patients with CHDMs in *TET2* had lower risk of major adverse cardiovascular events while taking canakinumab compared with placebo.^72^

Thirdly, a dose–response relationship between the variant allele frequency (VAF; a marker for clone size in which a VAF of 1% corresponds with mutations in 2% of leukocytes) and ASCVD is suggested.^2^, ^3^, ^44^, ^72^ Patients with a higher VAF had a higher coronary artery calcium score^2^ as well as a higher risk of major adverse cardiovascular events.^3^, ^44^ This dose‐dependent effect of the VAF was also suggested in patients with a genetic *IL6R* deficiency and *DNMT3A* or *TET2* CHDMs,^44^ because the effect of this genetic deficiency was mostly present in patients with a large clone size. As such, patients with a higher VAF may be better candidates for anti‐inflammatory therapy. Fourthly, although CHIP may cause atherosclerosis, atherosclerosis itself might also accelerate CH.^49^ *Apoe* ^*−/*−^ mice fed with an atherogenic diet showed increased HSC proliferation and increase in leukocytosis.^49^ Although this mouse model cannot exclude that the increase in HSC proliferation is primarily driven by the atherogenic diet itself, a second study in humans showed atherosclerosis increased proliferation markers in HSCs, whereas cholesterol levels in these patients were normal, suggesting increased proliferation and subsequent acceleration of CH^49^ (Figure 1).

Lastly, although the interaction between atherosclerosis and CHIP is increasingly established, most evidence published concerns CHDMs in *TET2*, *DNMT3A*, and *JAK2*.^2^, ^51^ Although in‐human association studies do suggest a proatherogenic effect of *DNMT3A* and *JAK2*, there are no publications on mouse studies that have clearly proven this to date.

In summary, the inflammasome/IL‐1/IL‐6 pathway plays a central role in CHIP‐induced ASCVD. Anti‐inflammatory therapies could, therefore, be an important asset to lower cardiovascular risk in patients with CH.

## CLONAL HEMATOPOIESIS ACROSS THE LEFT VENTRICLE EJECTION CLASSIFICATION

CHIP was first described as both an inducer and progressor of heart failure with an ischemic cause. However, later discoveries also depicted CHIP as a possible trigger of nonischemic heart failure with reduced ejection fraction (HFrEF), in the absence of any heart failure stimulant such as ischemia or an increased afterload (Figure 2).

## ISCHEMIC HFrEF

HFrEF originates from a lack of blood flow to the cardiomyocytes, most often secondary to atherosclerotic coronary artery disease. Although CH prevalence increases with age, its prevalence in ischemic HFrEF is not entirely attributable to patient age or CHIP detection techniques.^6^, ^7^, ^8^, ^9^, ^18^ CH associates with mortality in patients with HFrEF independent of age.^8^, ^9^, ^18^ Interestingly, mutations in genes other than *DNMT3A* and *TET2* may indicate increased risk for ischemic HFrEF.^6^, ^7^

CH doubles the risk of mortality or heart failure hospitalization in ischemic HFrEF,^8^, ^9^ but this is highly dependent on the specific gene mutation and the VAF. Using the current definition of CHIP (a VAF of at least 2%^1^), and including patients regardless of the mutated gene involved, CHIP increases the risk for cardiac adverse events (cardiac death and heart failure hospitalization) by a factor of 2.^8^, ^76^ Likewise, in a cardiogenic shock cohort (consisting of patients with ischemic and nonischemic HFrEF), CHIP doubled 30‐day mortality.^18^

The VAF cutoff at 2% to define CHIP was historically set by the technical sensitivity of exome sequencing.^3^, ^25^ Current technological advancements allow us to sequence even deeper below this threshold, even down to a VAF of 0.01%,^77^ and studies to date show promising results, providing more detailed clinical associations.^6^, ^8^, ^9^, ^77^ Already, sequencing up to a VAF of 0.5% showed that clone populations <2% are prognostically relevant in patients with ischemic HFrEF.^6^, ^8^, ^9^ Survival receiver operating characteristic curves show that *DNMT3A* and *TET2* mutations are prognostically relevant when clone size and corresponding VAF is at least 1.15% and 0.73%, respectively. Consequently, a new, lower cutoff value of VAF is suggested.^9^ Patients with a VAF above these thresholds had a 5‐year mortality rate of 31% to 32%, whereas below this threshold the rates were much lower at 18% to 19%.^9^ These results still need validation in larger, multicenter studies, but show much promise.

Like optimizing the VAF cutoff in a mutation‐specific manner, there are further indications of mutation‐led disease mechanisms. Studies thus far have implicated common inflammatory pathways, marked by different upstream regulators (Figure 3). There are currently no animal or human studies that directly compare different gene mutations in ischemic HFrEF. An overview of animal studies performed is provided in Table S1.

In mice with ischemic HFrEF and *Tet2*‐mutated CHDMs, *Tet2*‐deficient macrophages accumulate in the myocardium and atherosclerotic plaque,^11^ leading to a deterioration in cardiac function with lower ejection fraction and increased fibrosis. These macrophages show upregulation of the NLRP3‐inflammasome and increased IL‐1, IL‐6, and IL‐18 (interleukin‐18)^11^ expression. The mechanism by which IL‐6 is increased could also be NLRP3‐independent, because TET2 normally functions as an inhibitor of IL‐6 gene expression in the late phase of inflammation.^50^, ^78^ However, in mice with ischemic HFrEF and a *Jak2* ^V617F^ mutation,^12^ JAK/STAT signaling increased over time,^54^ leading to higher expression of IFN‐γ and increased AIM2 inflammasome activity.^54^ Inflammasome complexes consist of a sensor protein (eg, NLRP3 or AIM2), an adaptor protein, and an effector protein (ie, caspase‐1). In *Jak2* ^V617F^‐mutated macrophages, there seems to be an overactivation of the AIM2 sensor, as opposed to the NLRP3 sensor in *Dnmt3a*‐ and *Tet2*‐deficient macrophages.^11^, ^12^, ^16^ Similar to the activation of NLRP3, AIM2 also leads to the formation of the inflammasome complex that activates caspase‐1, allowing IL‐1 and IL‐18 maturation.^12^, ^54^ Therefore, although the proinflammatory outcome of *TET2* and *JAK2* mutations are the same, the upstream intracellular sensors used to form the activated inflammasome complexes are different (Figure 3). This suggests that the same drugs can be used to target the downstream proinflammatory cytokines, but for targeting upstream regulators (eg, by NLRP3, AIM2, or JAK2 inhibitors), the different sensor proteins must be considered. Both *Dnmt3a* and *Jak2* mutations led to a worse prognosis in mice, increasing cardiac inflammation and worsening cardiac fibrosis and function compared with their littermates without a mutation.^10^, ^12^ To date, mechanistic studies performed in patients used single‐cell RNA sequencing on peripheral blood mononuclear cells of patients with either ischemic HFrEF or aortic stenosis,^16,79^ and all had a *DNMT3A* or *TET2* CHDM. The NLRP3‐inflammasome/IL‐1/IL‐6 pathway was upregulated in circulating monocytes from patients with ischemic HFrEF.^16^ Future studies should correlate these findings to intracardiac inflammation and investigate the effect of NLRP3 (eg, colchicine), IL‐1 (eg, anakinra, canakinumab), or IL‐6 inhibition (eg, tocilizumab, ziltivekimab) in this patient population. An initial anti‐inflammatory study to prevent heart failure following myocardial infarction is already set but does not look at CH specifically (NCT05177822).

In summary, mice and patient studies provide evidence that targeting the inflammasome/IL‐1/IL‐6 pathway is worth exploring in the treatment of ischemic HFrEF. Future studies should investigate potential patient subgroups that would benefit from these immunotherapies, paving the way for the first clinical heart failure trial based primarily on CHIP.

## NONISCHEMIC HFrEF

Nonischemic HFrEF is a heterogeneous group of diseases that comprise dilated cardiomyopathy and hypokinetic nondilated cardiomyopathy. Coronary artery disease is excluded as a cause for nonischemic HFrEF, highlighting CH in an atherosclerosis‐independent manner. Interestingly, a population‐based analysis combining 5 non–heart failure cohorts shows CHIP predicts the incidence of heart failure, mainly in patients without previous ASCVD.^80^ CHIP increased the risk of subsequent onset of heart failure by 25%.^80^ Although data on coronary arteries and left ventricular ejection fraction status at time of heart failure onset are lacking, it is tempting to suggest CHIP predicts later onset of nonischemic HFrEF, especially because JAK2^V617F^ mutations are associated with reduced left ventricular ejection fraction.^80^

To date, 2 studies have analyzed the prognostic impact of CHIP in nonischemic HFrEF.^8^, ^18^ The first study comprised a relatively small population and detected CHIP in 24 of 62 patients with HFrEF, of which 12 had a nonischemic cause.^8^ The second study included patients with cardiogenic shock regardless of ischemic cause.^18^ CHIP doubled the risk for heart failure hospitalization or cardiac death, independent of an ischemic cause,^8^ and increased 30‐day mortality following cardiogenic shock.^18^ Although these results are promising and suggest a clear prognostic impact of CHIP on nonischemic HFrEF, they require validation in a larger population.

Mechanistically, CHIP worsens prognosis in HFrEF likely through the cardiac infiltration of immune cells (mainly monocytes) holding CHDMs (eg, in *TET2* or *DNMT3A*). Inflammation develops in the myocardium with subsequent reduction in systolic function and cardiac fibrosis (Figure 1). Therefore, several mechanisms that are present in nonischemic HFrEF are expected to be similar to the direct mechanisms of CHIP in ischemic HFrEF. Two mouse studies (using *Tet2* and *Jak2* ^V617F^ as CHDM genes) compared these 2 HFrEF causes. They showed that mice with either transverse constriction of the aorta (ie, pressure overload) or ligation of the anterior descending artery (ie, ischemia) had a comparable cardiac macrophage‐led inflammation profile with an inflammasome‐dependent immune response with IL‐1 and IL‐6,^11^, ^12^ strengthening the similarities between CHIP‐associated ischemic HFrEF and CHIP‐associated nonischemic HFrEF.

Most of the mechanistic studies on the effect of CHIP on heart failure used a trigger to simulate pressure overload (eg, transverse aortic constriction) or ischemia (eg, ligation of the anterior descending artery)^10^, ^11^, ^12^ in the mice. Importantly, when a bone marrow transplantation with *T*et2‐deficient hematopoietic stem cells was performed that led to *Tet2* CHIP, without using any trigger or conditioning to induce heart failure, HFrEF still developed.^14^ In this study, *Tet2*‐deficient macrophages also showed intracardiac upregulation of the inflammasome/IL‐1/IL‐6 pathway,^14^ suggesting that the innate immune system was overactive even in the absence of any trigger. This strongly contends that CHIP could be an inducer and accelerator of nonischemic HFrEF. One of the next steps is to translate anti‐inflammatory targets into the clinic. Current ongoing anti‐inflammatory trials do not yet subset patients based on CHIP (eg, NCT03797001, NCT04705987).

In summary, CHIP could be a novel therapeutic target in nonischemic HFrEF. Aiming to dampen this immune response through the application of anti‐inflammatory agents and other immunotherapies would open a new field to the HFrEF treatment regimen.

## HEART FAILURE WITH PRESERVED EJECTION FRACTION

Heart failure with preserved ejection fraction (HFpEF) is a highly complex, multiorgan syndrome, with multiple pathophysiological phenotypes.^81^, ^82^ Inflammation is highlighted as a key driver of the disease and a potential treatment target.^83^, ^84^, ^85^, ^86^, ^87^, ^88^, ^89^, ^90^, ^91^, ^92^ However, HFpEF has multiple patient phenogroups, and not every phenogroup is characterized by increased inflammation. CHIP may be of interest in at least some of the HFpEF phenogroups.

A recent publication underlines the potential role of CHIP in HFpEF. CHIP predicted the development of HFpEF in patients <65 years of age in a prospective population‐based cohort in Groningen, the Netherlands.^19^ CHIP was associated with a risk for HFpEF development twice as high as patients without CHIP and did not associate with an increased risk for HFrEF.^19^ Despite the age‐related nature of CHIP and association with comorbidities, CHIP may be a lone‐standing risk factor for HFpEF development below the age of 65 years.^19^ Additionally, CHIP predicts the onset of HF, whereas the most common CHDMs (ie, *DNMT3A* and *TET2*) could not predict reduction of left ventricular ejection fraction, suggesting CHIP predicts onset of HFpEF in at least a minority of patients with CHIP.^80^

No other human studies on HFpEF and CHIP have been performed to date, and even CHIP mouse models always led to a HFrEF phenotype^10^, ^11^, ^12^ (Figure 2 and Table S1). Nevertheless, multiple HFpEF studies did report inflammatory profiles with striking similarity to inflammatory pathways upregulated in CHIP^86^, ^89^, ^90^, ^91^, ^93^, ^94^ (Table). In particular, soluble IL‐1 receptor, IL‐6, and C‐reactive protein were upregulated and correlated with a worse prognosis in HFpEF,^89^, ^91^ which also associated with the inflammasome/IL‐1/IL‐6 pathway in HFrEF and CHIP.^10^, ^14^, ^16^ Surprisingly, the expression of these CHIP‐associated biomarkers was even higher in HFpEF compared with HFrEF.^89^ A clinical trial to suppress NLRP3 inflammasome activity using colchicine is currently ongoing in HFpEF (NCT04857931). Additionally, the tumor necrosis factor family was upregulated in a subgroup of patients with HFpEF with multiple comorbidities (eg, obesity, diabetes).^91^, ^94^ TNF‐α itself drives clonal expansion with myeloid skewing at least in an in vitro setting,^95^ and an increase in TNF‐α was also observed in circulating monocytes of patients with heart failure (including aortic stenosis patients) with *DNTM3A* CHDMs, as well as in pressure‐overload mice models with *Jak2* CHDMs.^12^, ^79^ Although previous TNF‐α trials did not improve outcome in patients with HFrEF,^96^, ^97^ better patient selection based on CHIP could help to identify a targetable patient subgroup for this treatment.

**Table:** Clinical Studies Showing the Inflammasome/IL‐1/IL‐6 Pathway Is Often Upregulated in HFpEF

First author (year)	No. of HFpEF patients	No. of control patients	Type of controls	Increase of CH‐associated cytokines in HFpEF	Comment
Matsubara^90^ (2011)	82	171	Patients without HF or another type of HF	Yes	CRP and IL‐6 were upregulated in HFpEF.
Santhanakrishnan^86^ (2012)	50	101	Patients without HF or another type of HF	No	il1rl1 was tested and was not increased in the HFpEF study group, possible due to small sample sizes.
Sanders‐van Wijk^89^ (2015)	112	458	Patients with another type of HF	Yes	il1rl1, hs‐CRP, and IL‐6 were upregulated in HFpEF.
Van Tassell^93^ (2018)	21	10	Patients with HFpEF who were not treated with IL‐1 blockade	Yes	IL‐1 blockade by anakinra reduced CRP and NT‐proBNP in HFpEF.
Sanders‐van Wijk^94^ (2020)	345	30	Patients without HF or another type of HF	Yes	IL‐1, IL‐6, and TNF‐α were upregulated in 2 separate clusters of HFpEF patients.
Kresoja^91^ (2021)	999	999	Patients without HF	Yes	IL‐1, the TNF superfamily, and IL‐6 were upregulated in HFpEF.

CH indicates clonal hematopoiesis; CRP, C‐reactive protein; HF, heart failure; HFpEF, heart failure with preserved ejection fraction; hs‐CRP, high‐sensitivity C‐reactive protein; IL‐1, interleukin‐1; il1rl1, interleukin 1 receptor ligand‐1; IL‐6, interleukin‐6; TNF, tumor necrosis factor; and TNF‐α, tumor necrosis factor‐α.

In summary, research has begun to show associations between CHIP and HFpEF development, but any underlying pathophysiological mechanism is still speculative. Previous studies investigating inflammation in HFpEF highlight overlapping biomarker profiles with CHIP.

## FUTURE OUTLOOK AND POTENTIAL TREATMENT TARGETS

CHIP could be an important contributor to our understanding of the phenotypes of inflammatory heart failure, both as a diagnostic marker and a treatment guide. A feedback loop may exist between inflammation and CHIP, which deserves further investigation in a heart failure setting. Heart failure is associated with elevated circulating proinflammatory cytokines,^98^ and these cytokines (TNF‐α, IL‐1) stimulate CHIP at least in mice and in vitro in humans.^61^, ^95^ Even when this feedback loop does not exist, CHIP is still a biomarker for heart failure development and progression, and mechanistic studies performed already suggest a benefit in targeting the associated inflammatory pathways (Figure 3).

The evidence accumulated over the past several years is sufficient to initiate a clinical trial that targets CHIP. Firstly, proinflammatory cytokines downstream of *TET2*, *DNMT3A*, and *JAK2* ^*V617F*^ CHDMs are targetable with immunotherapy, as revealed in both murine and human studies. Secondly, in a single‐center study, the variant allele frequency of *TET2* and *DNMT3A* CHDMs was suggested to be already clinically significant at 0.73% and 1.15%, respectively (no clinical heart failure study has been performed on *JAK2* ^*V617F*^). Thirdly, both *DNMT3A* and *TET2* CHDMs have been associated with the same targetable upstream sensor protein NLRP3, initiating the inflammasome/IL‐1/IL‐6 cascade. This does not count for *JAK2* ^*V617F*^ mutations, which are associated with AIM2.^12^, ^54^ Finally, there are already promising data on the use of an IL‐1 blockade to prevent major adverse cardiovascular events in patients^72^, ^75^ with *TET2* mutations.^72^ Therefore, it would be possible and arguably vital to initiate a clinical trial with NLRP3, IL‐1, or IL‐6 blockers with patients who have *DNMT3A* and/or *TET2* CHDMs, and a variant allele frequency of 1.15% or 0.73%, respectively. There are already 2 studies registered as clinical trials that target CHIP, a phase I study on selnoflast (ie, a NLRP3 inhibitor) in patient CHDMs in *TET2* and ASCVD (10 520 571 in the International Traditional Medicine Clinical Trial Registry), and a phase II study on colchicine in patients with CHIP and ischemic HFrEF (2021‐001508‐13 in the European Union Clinical Trials Register). No clinical trials on nonischemic HFrEF have been registered to date.

Lastly, although our review focusses on CHIP, mosaic loss of the Y chromosome is another blood disorder comparable to CHIP of potential future interest for heart failure specialists and deserves mentioning. Mosaic loss of the Y chromosome, a common blood disorder in men in which a proportion of white blood cells lose their Y chromosome, leads to the onset of nonischemic HFrEF in mice, possibly via dysfunctional macrophages that release tumor growth factor‐β1 and trigger myocardial fibrosis.^99^ Mosaic loss of the Y chromosome is already associated with increased mortality after transcatheter aortic valve implantation for aortic stenosis.^100^

## CONCLUSIONS

CHIP is a contributor to heart failure development regardless of ejection fraction phenotype. The discovery and improved mechanistic understanding of this phenomenon provide the possibility to select patients who will benefit from new immunotherapies in this novel area of heart failure therapeutics. Basic and translational research should work in parallel to discover gene‐specific disease mechanisms and identify new patient subgroups potentially eligible for immunotherapy.

## Sources of Funding

This work was funded by the Dutch Cardiovascular Alliance, an initiative with support of the Dutch Heart Foundation, and Stichting Hartedroom for financing the Double Dose program 2020B005 (principal investigator: S.R.B.H.). J.A.J.V. is supported by a research grant from the Dutch Heart Foundation.

## Disclosures

None.

## Supporting information

Table S1

## References

[1] Steensma DP , Bejar R , Jaiswal S , Lindsley RC , Sekeres MA , Hasserjian RP , Ebert BL . Clonal hematopoiesis of indeterminate potential and its distinction from myelodysplastic syndromes. Blood. 2015;126:9–16. doi: 10.1182/blood-2015-03-631747 25931582 PMC4624443

[2] Jaiswal S , Natarajan P , Silver AJ , Gibson CJ , Bick AG , Shvartz E , McConkey M , Gupta N , Gabriel S , Ardissino D , et al. Clonal hematopoiesis and risk of atherosclerotic cardiovascular disease. N Engl J Med. 2017;377:111–121. doi: 10.1056/NEJMoa1701719 28636844 PMC6717509

[3] Jaiswal S , Fontanillas P , Flannick J , Manning A , Grauman PV , Mar BG , Lindsley RC , Mermel CH , Burtt N , Chavez A , et al. Age‐related clonal hematopoiesis associated with adverse outcomes. N Engl J Med. 2014;371:2488–2498. doi: 10.1056/NEJMoa1408617 25426837 PMC4306669

[4] Genovese G , Kähler AK , Handsaker RE , Lindberg J , Rose SA , Bakhoum SF , Chambert K , Mick E , Neale BM , Fromer M , et al. Clonal hematopoiesis and blood‐cancer risk inferred from blood DNA sequence. N Engl J Med. 2014;371:2477–2487. doi: 10.1056/NEJMoa1409405 25426838 PMC4290021

[5] Nachun D , Lu AT , Bick AG , Natarajan P , Weinstock J , Szeto MD , Kathiresan S , Abecasis G , Taylor KD , Guo X , et al. Clonal hematopoiesis associated with epigenetic aging and clinical outcomes. Aging Cell. 2021;20:e13366. doi: 10.1111/acel.13366 34050697 PMC8208788

[6] Kiefer KC , Cremer S , Pardali E , Assmus B , Abou‐El‐Ardat K , Kirschbaum K , Dorsheimer L , Rasper T , Berkowitsch A , Serve H , et al. Full spectrum of clonal haematopoiesis‐driver mutations in chronic heart failure and their associations with mortality. ESC Heart Fail. 2021;8:1873–1884. doi: 10.1002/ehf2.13297 33779075 PMC8120376

[7] Cremer S , Kirschbaum K , Berkowitsch A , John D , Kiefer K , Dorsheimer L , Wagner J , Rasper T , Abou‐El‐Ardat K , Assmus B , et al. Multiple somatic mutations for clonal hematopoiesis are associated with increased mortality in patients with chronic heart failure. Circ Genom Precis Med. 2020;13:e003003. doi: 10.1161/circgen.120.003003 32598856

[8] Pascual‐Figal DA , Bayes‐Genis A , Díez‐Díez M , Hernández‐Vicente Á , Vázquez‐Andrés D , de la Barrera J , Vazquez E , Quintas A , Zuriaga MA , Asensio‐López MC , et al. Clonal hematopoiesis and risk of progression of heart failure with reduced left ventricular ejection fraction. J Am Coll Cardiol. 2021;77:1747–1759. doi: 10.1016/j.jacc.2021.02.028 33832602

[9] Assmus B , Cremer S , Kirschbaum K , Culmann D , Kiefer K , Dorsheimer L , Rasper T , Abou‐El‐Ardat K , Herrmann E , Berkowitsch A , et al. Clonal haematopoiesis in chronic ischaemic heart failure: prognostic role of clone size for DNMT3A‐ and TET2‐driver gene mutations. Eur Heart J. 2021;42:257–265. doi: 10.1093/eurheartj/ehaa845 33241418

[10] Sano S , Oshima K , Wang Y , Katanasaka Y , Sano M , Walsh K . CRISPR‐mediated gene editing to assess the roles of Tet2 and Dnmt3a in clonal hematopoiesis and cardiovascular disease. Circ Res. 2018;123:335–341. doi: 10.1161/circresaha.118.313225 29728415 PMC6054544

[11] Sano S , Oshima K , Wang Y , MacLauchlan S , Katanasaka Y , Sano M , Zuriaga MA , Yoshiyama M , Goukassian D , Cooper MA , et al. Tet2‐mediated clonal hematopoiesis accelerates heart failure through a mechanism involving the IL‐1β/NLRP3 inflammasome. J Am Coll Cardiol. 2018;71:875–886. doi: 10.1016/j.jacc.2017.12.037 29471939 PMC5828038

[12] Sano S , Wang Y , Yura Y , Sano M , Oshima K , Yang Y , Katanasaka Y , Min KD , Matsuura S , Ravid K , et al. JAK2 (V617F) ‐mediated clonal hematopoiesis accelerates pathological remodeling in murine heart failure. JACC Basic Transl Sci. 2019;4:684–697. doi: 10.1016/j.jacbts.2019.05.013 31709318 PMC6834960

[13] Sano S , Wang Y , Ogawa H , Horitani K , Sano M , Polizio AH , Kour A , Yura Y , Doviak H , Walsh K . TP53‐mediated therapy‐related clonal hematopoiesis contributes to doxorubicin‐induced cardiomyopathy by augmenting a neutrophil‐mediated cytotoxic response. JCI Insight. 2021;6:e146076. doi: 10.1172/jci.insight.146076 34236050 PMC8410064

[14] Wang Y , Sano S , Yura Y , Ke Z , Sano M , Oshima K , Ogawa H , Horitani K , Min KD , Miura‐Yura E , et al. Tet2‐mediated clonal hematopoiesis in nonconditioned mice accelerates age‐associated cardiac dysfunction. JCI Insight. 2020;5:e135204. doi: 10.1172/jci.insight.135204 32154790 PMC7213793

[15] Yura Y , Miura‐Yura E , Katanasaka Y , Min KD , Chavkin N , Polizio AH , Ogawa H , Horitani K , Doviak H , Evans MA , et al. The cancer therapy‐related clonal hematopoiesis driver gene Ppm1d promotes inflammation and non‐ischemic heart failure in mice. Circ Res. 2021;129:684–698. doi: 10.1161/circresaha.121.319314 34315245 PMC8409899

[16] Abplanalp WT , Cremer S , John D , Hoffmann J , Schuhmacher B , Merten M , Rieger MA , Vasa‐Nicotera M , Zeiher AM , Dimmeler S . Clonal hematopoiesis‐driver DNMT3A mutations alter immune cells in heart failure. Circ Res. 2021;128:216–228. doi: 10.1161/circresaha.120.317104 33155517

[17] Mas‐Peiro S , Hoffmann J , Fichtlscherer S , Dorsheimer L , Rieger MA , Dimmeler S , Vasa‐Nicotera M , Zeiher AM . Clonal haematopoiesis in patients with degenerative aortic valve stenosis undergoing transcatheter aortic valve implantation. Eur Heart J. 2020;41:933–939. doi: 10.1093/eurheartj/ehz591 31504400 PMC7033916

[18] Scolari FL , Abelson S , Brahmbhatt DH , Medeiros JJF , Fan CS , Fung NL , Mihajlovic V , Anker MS , Otsuki M , Lawler PR , et al. Clonal haematopoiesis is associated with higher mortality in patients with cardiogenic shock. Eur J Heart Fail. 2022;24:1573–1582. doi: 10.1002/ejhf.2588 35729851

[19] Shi C , Aboumsallem JP , Suthahar N , de Graaf AO , Jansen JH , van Zeventer IA , Bracun V , de Wit S , Screever EM , van den Berg PF , et al. Clonal haematopoiesis of indeterminate potential: associations with heart failure incidence, clinical parameters and biomarkers. Eur J Heart Fail. 2022;25:4–13. doi: 10.1002/ejhf.2715 36221810 PMC10092539

[20] Fuster JJ , Walsh K . Somatic mutations and clonal hematopoiesis: unexpected potential new drivers of age‐related cardiovascular disease. Circ Res. 2018;122:523–532. doi: 10.1161/circresaha.117.312115 29420212 PMC5826570

[21] Bhattacharya R , Bick AG . Clonal hematopoiesis of indeterminate potential: an expanding genetic cause of cardiovascular disease. Curr Atheroscler Rep. 2021;23:66. doi: 10.1007/s11883-021-00966-9 34468876 PMC8543762

[22] Jaiswal S , Libby P . Clonal haematopoiesis: connecting ageing and inflammation in cardiovascular disease. Nat Rev Cardiol. 2020;17:137–144. doi: 10.1038/s41569-019-0247-5 31406340 PMC9448847

[23] Sano S , Wang Y , Walsh K . Clonal hematopoiesis and its impact on cardiovascular disease. Circ J. 2018;83:2–11. doi: 10.1253/circj.CJ-18-0871 30185689 PMC6310086

[24] Chavakis T , Wielockx B , Hajishengallis G . Inflammatory modulation of hematopoiesis: linking trained immunity and clonal hematopoiesis with chronic disorders. Annu Rev Physiol. 2022;84:183–207. doi: 10.1146/annurev-physiol-052521-013627 34614373

[25] Jaiswal S , Ebert BL . Clonal hematopoiesis in human aging and disease. Science. 2019;366:eaan4673. doi: 10.1126/science.aan4673 31672865 PMC8050831

[26] Natarajan P , Jaiswal S , Kathiresan S . Clonal hematopoiesis: somatic mutations in blood cells and atherosclerosis. Circ Genom Precis Med. 2018;11:e001926. doi: 10.1161/circgen.118.001926 29987111 PMC6082163

[27] Khetarpal SA , Qamar A , Bick AG , Fuster JJ , Kathiresan S , Jaiswal S , Natarajan P . Clonal hematopoiesis of indeterminate potential reshapes age‐related CVD: JACC review topic of the week. J Am Coll Cardiol. 2019;74:578–586. doi: 10.1016/j.jacc.2019.05.045 31345433 PMC6662618

[28] Challen GA , Goodell MA . Clonal hematopoiesis: mechanisms driving dominance of stem cell clones. Blood. 2020;136:1590–1598. doi: 10.1182/blood.2020006510 32746453 PMC7530644

[29] Rossi L , Lin KK , Boles NC , Yang L , King KY , Jeong M , Mayle A , Goodell MA . Less is more: unveiling the functional core of hematopoietic stem cells through knockout mice. Cell Stem Cell. 2012;11:302–317. doi: 10.1016/j.stem.2012.08.006 22958929 PMC3461270

[30] Avagyan S , Henninger JE , Mannherz WP , Mistry M , Yoon J , Yang S , Weber MC , Moore JL , Zon LI . Resistance to inflammation underlies enhanced fitness in clonal hematopoiesis. Science. 2021;374:768–772. doi: 10.1126/science.aba9304 34735227

[31] Cooper JN , Young NS . Clonality in context: hematopoietic clones in their marrow environment. Blood. 2017;130:2363–2372. doi: 10.1182/blood-2017-07-794362 29046282 PMC5709788

[32] Jeong M , Park HJ , Celik H , Ostrander EL , Reyes JM , Guzman A , Rodriguez B , Lei Y , Lee Y , Ding L , et al. Loss of Dnmt3a immortalizes hematopoietic stem cells in vivo. Cell Rep. 2018;23:1–10. doi: 10.1016/j.celrep.2018.03.025 29617651 PMC5908249

[33] Cimmino L , Dolgalev I , Wang Y , Yoshimi A , Martin GH , Wang J , Ng V , Xia B , Witkowski MT , Mitchell‐Flack M , et al. Restoration of TET2 function blocks aberrant self‐renewal and leukemia progression. Cell. 2017;170:1079–1095.e1020. doi: 10.1016/j.cell.2017.07.032 28823558 PMC5755977

[34] Bolton KL , Ptashkin RN , Gao T , Braunstein L , Devlin SM , Kelly D , Patel M , Berthon A , Syed A , Yabe M , et al. Cancer therapy shapes the fitness landscape of clonal hematopoiesis. Nat Genet. 2020;52:1219–1226. doi: 10.1038/s41588-020-00710-0 33106634 PMC7891089

[35] Wong TN , Ramsingh G , Young AL , Miller CA , Touma W , Welch JS , Lamprecht TL , Shen D , Hundal J , Fulton RS , et al. Role of TP53 mutations in the origin and evolution of therapy‐related acute myeloid leukaemia. Nature. 2015;518:552–555. doi: 10.1038/nature13968 25487151 PMC4403236

[36] Kahn JD , Miller PG , Silver AJ , Sellar RS , Bhatt S , Gibson C , McConkey M , Adams D , Mar B , Mertins P , et al. PPM1D‐truncating mutations confer resistance to chemotherapy and sensitivity to PPM1D inhibition in hematopoietic cells. Blood. 2018;132:1095–1105. doi: 10.1182/blood-2018-05-850339 29954749 PMC6137556

[37] Xie M , Lu C , Wang J , McLellan MD , Johnson KJ , Wendl MC , McMichael JF , Schmidt HK , Yellapantula V , Miller CA , et al. Age‐related mutations associated with clonal hematopoietic expansion and malignancies. Nat Med. 2014;20:1472–1478. doi: 10.1038/nm.3733 25326804 PMC4313872

[38] Jeong M , Sun D , Luo M , Huang Y , Challen GA , Rodriguez B , Zhang X , Chavez L , Wang H , Hannah R , et al. Large conserved domains of low DNA methylation maintained by Dnmt3a. Nat Genet. 2014;46:17–23. doi: 10.1038/ng.2836 24270360 PMC3920905

[39] Challen GA , Sun D , Jeong M , Luo M , Jelinek J , Berg JS , Bock C , Vasanthakumar A , Gu H , Xi Y , et al. Dnmt3a is essential for hematopoietic stem cell differentiation. Nat Genet. 2011;44:23–31. doi: 10.1038/ng.1009 22138693 PMC3637952

[40] Drake JW . A constant rate of spontaneous mutation in DNA‐based microbes. Proc Natl Acad Sci USA. 1991;88:7160–7164. doi: 10.1073/pnas.88.16.7160 1831267 PMC52253

[41] Jaiswal S . Clonal hematopoiesis and nonhematologic disorders. Blood. 2020;136:1606–1614. doi: 10.1182/blood.2019000989 32736379 PMC8209629

[42] Yahata T , Takanashi T , Muguruma Y , Ibrahim AA , Matsuzawa H , Uno T , Sheng Y , Onizuka M , Ito M , Kato S , et al. Accumulation of oxidative DNA damage restricts the self‐renewal capacity of human hematopoietic stem cells. Blood. 2011;118:2941–2950. doi: 10.1182/blood-2011-01-330050 21734240

[43] Dawoud AAZ , Tapper WJ , Cross NCP . Clonal myelopoiesis in the UK Biobank cohort: ASXL1 mutations are strongly associated with smoking. Leukemia. 2020;34:2660–2672. doi: 10.1038/s41375-020-0896-8 32518416

[44] Bick AG , Pirruccello JP , Griffin GK , Gupta N , Gabriel S , Saleheen D , Libby P , Kathiresan S , Natarajan P . Genetic interleukin 6 signaling deficiency attenuates cardiovascular risk in clonal hematopoiesis. Circulation. 2020;141:124–131. doi: 10.1161/circulationaha.119.044362 31707836 PMC7008855

[45] Hormaechea‐Agulla D , Matatall KA , Le DT , Kain B , Long X , Kus P , Jaksik R , Challen GA , Kimmel M , King KY . Chronic infection drives Dnmt3a‐loss‐of‐function clonal hematopoiesis via IFNγ signaling. Cell Stem Cell. 2021;28:1428–1442. doi: 10.1016/j.stem.2021.03.002 33743191 PMC8349829

[46] van der Heijden WA , van Deuren RC , van de Wijer L , van den Munckhof ICL , Steehouwer M , Riksen NP , Netea MG , de Mast Q , Vandekerckhove L , de Voer RM , et al. Clonal hematopoiesis is associated with low CD4 nadir and increased residual HIV transcriptional activity in virally suppressed individuals with HIV. J Infect Dis. 2021;225:1339–1347. doi: 10.1093/infdis/jiab419 PMC9016425 34417800

[47] Bick AG , Weinstock JS , Nandakumar SK , Fulco CP , Bao EL , Zekavat SM , Szeto MD , Liao X , Leventhal MJ , Nasser J , et al. Inherited causes of clonal haematopoiesis in 97,691 whole genomes. Nature. 2020;586:763–768. doi: 10.1038/s41586-020-2819-2 33057201 PMC7944936

[48] Bao EL , Nandakumar SK , Liao X , Bick AG , Karjalainen J , Tabaka M , Gan OI , Havulinna AS , Kiiskinen TTJ , Lareau CA , et al. Inherited myeloproliferative neoplasm risk affects haematopoietic stem cells. Nature. 2020;586:769–775. doi: 10.1038/s41586-020-2786-7 33057200 PMC7606745

[49] Heyde A , Rohde D , McAlpine CS , Zhang S , Hoyer FF , Gerold JM , Cheek D , Iwamoto Y , Schloss MJ , Vandoorne K , et al. Increased stem cell proliferation in atherosclerosis accelerates clonal hematopoiesis. Cell. 2021;184:1348–1361.e1322. doi: 10.1016/j.cell.2021.01.049 33636128 PMC8109274

[50] Zhang Q , Zhao K , Shen Q , Han Y , Gu Y , Li X , Zhao D , Liu Y , Wang C , Zhang X , et al. Tet2 is required to resolve inflammation by recruiting Hdac2 to specifically repress IL‐6. Nature. 2015;525:389–393. doi: 10.1038/nature15252 26287468 PMC4697747

[51] Fuster JJ , MacLauchlan S , Zuriaga MA , Polackal MN , Ostriker AC , Chakraborty R , Wu CL , Sano S , Muralidharan S , Rius C , et al. Clonal hematopoiesis associated with TET2 deficiency accelerates atherosclerosis development in mice. Science. 2017;355:842–847. doi: 10.1126/science.aag1381 28104796 PMC5542057

[52] Liao M , Chen R , Yang Y , He H , Xu L , Jiang Y , Guo Z , He W , Jiang H , Wang J . Aging‐elevated inflammation promotes DNMT3A R878H‐driven clonal hematopoiesis. Acta Pharm Sin B. 2022;12:678–691. doi: 10.1016/j.apsb.2021.09.015 35256939 PMC8897035

[53] Cordua S , Kjaer L , Skov V , Pallisgaard N , Hasselbalch HC , Ellervik C . Prevalence and phenotypes of JAK2 V617F and calreticulin mutations in a Danish general population. Blood. 2019;134:469–479. doi: 10.1182/blood.2019001113 31217187

[54] Fidler TP , Xue C , Yalcinkaya M , Hardaway B , Abramowicz S , Xiao T , Liu W , Thomas DG , Hajebrahimi MA , Pircher J , et al. The AIM2 inflammasome exacerbates atherosclerosis in clonal haematopoiesis. Nature. 2021;592:296–301. doi: 10.1038/s41586-021-03341-5 33731931 PMC8038646

[55] Wolach O , Sellar RS , Martinod K , Cherpokova D , McConkey M , Chappell RJ , Silver AJ , Adams D , Castellano CA , Schneider RK , et al. Increased neutrophil extracellular trap formation promotes thrombosis in myeloproliferative neoplasms. Sci Transl Med. 2018;10:eaan8292. doi: 10.1126/scitranslmed.aan8292 29643232 PMC6442466

[56] Landolfi R , Marchioli R , Kutti J , Gisslinger H , Tognoni G , Patrono C , Barbui T . Efficacy and safety of low‐dose aspirin in polycythemia vera. N Engl J Med. 2004;350:114–124. doi: 10.1056/NEJMoa035572 14711910

[57] Levin MG , Nakao T , Zekavat SM , Koyama S , Bick AG , Niroula A , Ebert B , Damrauer SM , Natarajan P . Genetics of smoking and risk of clonal hematopoiesis. Sci Rep. 2022;12:7248. doi: 10.1038/s41598-022-09604-z 35508625 PMC9068754

[58] Andersson‐Assarsson JC , van Deuren RC , Kristensson FM , Steehouwer M , Sjöholm K , Svensson PA , Pieterse M , Gilissen C , Taube M , Jacobson P , et al. Evolution of age‐related mutation‐driven clonal haematopoiesis over 20 years is associated with metabolic dysfunction in obesity. EBioMedicine. 2023;92:104621. doi: 10.1016/j.ebiom.2023.104621 37209535 PMC10209127

[59] Tercan H , Van Deuren RC , Schraa K , Horst RT , Van Den Munckhof IC , Bekkering S , Rutten JH , Netea MG , Joosten LAB , Hoischen A , et al. Clonal hematopoiesis and inflammation in obesity. Atherosclerosis. 2021;331:E113. doi: 10.1016/j.atherosclerosis.2021.06.333

[60] Fuster JJ , Zuriaga MA , Zorita V , MacLauchlan S , Polackal MN , Viana‐Huete V , Ferrer‐Pérez A , Matesanz N , Herrero‐Cervera A , Sano S , et al. TET2‐loss‐of‐function‐driven clonal hematopoiesis exacerbates experimental insulin resistance in aging and obesity. Cell Rep. 2020;33:108326. doi: 10.1016/j.celrep.2020.108326 33113366 PMC7856871

[61] Caiado F , Kovtonyuk LV , Gonullu NG , Fullin J , Boettcher S , Manz MG . Aging drives Tet2+/− clonal hematopoiesis via IL‐1 signaling. Blood. 2022;141:886–903. doi: 10.1182/blood.2022016835 36379023

[62] Pasupuleti SK , Ramdas B , Burns SS , Palam LR , Kanumuri R , Kumar R , Pandhiri TR , Dave U , Yellapu NK , Zhou X , et al. Obesity induced inflammation exacerbates clonal hematopoiesis. J Clin Invest. 2023;133:e163968. doi: 10.1172/jci163968 37071471 PMC10231999

[63] Yvan‐Charvet L , Pagler T , Gautier EL , Avagyan S , Siry RL , Han S , Welch CL , Wang N , Randolph GJ , Snoeck HW , et al. ATP‐binding cassette transporters and HDL suppress hematopoietic stem cell proliferation. Science. 2010;328:1689–1693. doi: 10.1126/science.1189731 20488992 PMC3032591

[64] Gu Q , Yang X , Lv J , Zhang J , Xia B , Kim JD , Wang R , Xiong F , Meng S , Clements TP , et al. AIBP‐mediated cholesterol efflux instructs hematopoietic stem and progenitor cell fate. Science. 2019;363:1085–1088. doi: 10.1126/science.aav1749 30705153 PMC6469354

[65] Sánchez‐Cabo F , Fuster JJ . Clonal haematopoiesis and atherosclerosis: a chicken or egg question? Nat Rev Cardiol. 2021;18:463–464. doi: 10.1038/s41569-021-00554-z 33859398

[66] Haring B , Reiner AP , Liu J , Tobias DK , Whitsel E , Berger JS , Desai P , Wassertheil‐Smoller S , LaMonte MJ , Hayden KM , et al. Healthy lifestyle and clonal hematopoiesis of indeterminate potential: results from the Women's Health Initiative. J Am Heart Assoc. 2021;10:e018789. doi: 10.1161/jaha.120.018789 33619969 PMC8174283

[67] Miller P , Qiao D , Rojas‐Quintero J , Honigberg MC , Sperling AS , Gibson CJ , Bick AG , Niroula A , McConkey ME , Sandoval B , et al. Association of clonal hematopoiesis with chronic obstructive pulmonary disease. Blood. 2021;139:357–368. doi: 10.1182/blood.2021013531 PMC8777202 34855941

[68] Zink F , Stacey SN , Norddahl GL , Frigge ML , Magnusson OT , Jonsdottir I , Thorgeirsson TE , Sigurdsson A , Gudjonsson SA , Gudmundsson J , et al. Clonal hematopoiesis, with and without candidate driver mutations, is common in the elderly. Blood. 2017;130:742–752. doi: 10.1182/blood-2017-02-769869 28483762 PMC5553576

[69] Coombs CC , Zehir A , Devlin SM , Kishtagari A , Syed A , Jonsson P , Hyman DM , Solit DB , Robson ME , Baselga J , et al. Therapy‐related clonal hematopoiesis in patients with non‐hematologic cancers is common and associated with adverse clinical outcomes. Cell Stem Cell. 2017;21:374–382.e374. doi: 10.1016/j.stem.2017.07.010 28803919 PMC5591073

[70] Pedersen KM , Çolak Y , Ellervik C , Hasselbalch HC , Bojesen SE , Nordestgaard BG . Smoking and increased white and red blood cells. Arterioscler Thromb Vasc Biol. 2019;39:965–977. doi: 10.1161/atvbaha.118.312338 30866659

[71] Wang S , Hu S , Luo X , Bao X , Li J , Liu M , Lv Y , Zhao C , Zeng M , Chen X , et al. Prevalence and prognostic significance of DNMT3A‐ and TET2‐ clonal haematopoiesis‐driver mutations in patients presenting with ST‐segment elevation myocardial infarction. EBioMedicine. 2022;78:103964. doi: 10.1016/j.ebiom.2022.103964 35339897 PMC8960977

[72] Svensson EC , Madar A , Campbell CD , He Y , Sultan M , Healey ML , Xu H , D'Aco K , Fernandez A , Wache‐Mainier C , et al. TET2‐driven clonal hematopoiesis and response to canakinumab: an exploratory analysis of the CANTOS randomized clinical trial. JAMA Cardiol. 2022;7:521–528. doi: 10.1001/jamacardio.2022.0386 35385050 PMC8988022

[73] Vlasschaert C , Heimlich JB , Rauh MJ , Natarajan P , Bick AG . Interleukin‐6 receptor polymorphism attenuates clonal hematopoiesis‐mediated coronary artery disease risk among 451 180 individuals in the UK Biobank. Circulation. 2023;147:358–360. doi: 10.1161/circulationaha.122.062126 36689568 PMC9883044

[74] Arends CM , Liman TG , Strzelecka PM , Kufner A , Löwe P , Huo S , Stein CM , Piper SK , Tilgner M , Sperber PS , et al. Associations of clonal hematopoiesis with recurrent vascular events and death in patients with incident ischemic stroke. Blood. 2023;141:787–799. doi: 10.1182/blood.2022017661 36441964

[75] Ridker PM , Everett BM , Thuren T , MacFadyen JG , Chang WH , Ballantyne C , Fonseca F , Nicolau J , Koenig W , Anker SD , et al. Antiinflammatory therapy with canakinumab for atherosclerotic disease. N Engl J Med. 2017;377:1119–1131. doi: 10.1056/NEJMoa1707914 28845751

[76] Dorsheimer L , Assmus B , Rasper T , Ortmann CA , Ecke A , Abou‐El‐Ardat K , Schmid T , Brüne B , Wagner S , Serve H , et al. Association of mutations contributing to clonal hematopoiesis with prognosis in chronic ischemic heart failure. JAMA Cardiol. 2019;4:25–33. doi: 10.1001/jamacardio.2018.3965 30566180 PMC6439691

[77] Acuna‐Hidalgo R , Sengul H , Steehouwer M , van de Vorst M , Vermeulen SH , Kiemeney L , Veltman JA , Gilissen C , Hoischen A . Ultra‐sensitive sequencing identifies high prevalence of clonal hematopoiesis‐associated mutations throughout adult life. Am J Hum Genet. 2017;101:50–64. doi: 10.1016/j.ajhg.2017.05.013 28669404 PMC5501773

[78] Cull AH , Snetsinger B , Buckstein R , Wells RA , Rauh MJ . Tet2 restrains inflammatory gene expression in macrophages. Exp Hematol. 2017;55:56–70.e13. doi: 10.1016/j.exphem.2017.08.001 28826859

[79] Abplanalp WT , Mas‐Peiro S , Cremer S , John D , Dimmeler S , Zeiher AM . Association of clonal hematopoiesis of indeterminate potential with inflammatory gene expression in patients with severe degenerative aortic valve stenosis or chronic postischemic heart failure. JAMA Cardiol. 2020;5:1170–1175. doi: 10.1001/jamacardio.2020.2468 32639511 PMC7344831

[80] Yu B , Roberts MB , Raffield LM , Zekavat SM , Nguyen NQH , Biggs ML , Brown MR , Griffin G , Desai P , Correa A , et al. Supplemental association of clonal hematopoiesis with incident heart failure. J Am Coll Cardiol. 2021;78:42–52. doi: 10.1016/j.jacc.2021.04.085 34210413 PMC8313294

[81] Pieske B , Tschöpe C , de Boer RA , Fraser AG , Anker SD , Donal E , Edelmann F , Fu M , Guazzi M , Lam CSP , et al. How to diagnose heart failure with preserved ejection fraction: the HFA‐PEFF diagnostic algorithm: a consensus recommendation from the Heart Failure Association (HFA) of the European Society of Cardiology (ESC). Eur Heart J. 2019;40:3297–3317. doi: 10.1093/eurheartj/ehz641 31504452

[82] McDonagh TA , Metra M , Adamo M , Gardner RS , Baumbach A , Böhm M , Burri H , Butler J , Čelutkienė J , Chioncel O , et al. 2021 ESC guidelines for the diagnosis and treatment of acute and chronic heart failure. Eur Heart J. 2021;42:3599–3726. doi: 10.1093/eurheartj/ehab368 34447992

[83] van Empel V , Brunner‐La Rocca HP . Inflammation in HFpEF: key or circumstantial? Int J Cardiol. 2015;189:259–263. doi: 10.1016/j.ijcard.2015.04.110 25897922

[84] Simmonds SJ , Cuijpers I , Heymans S , Jones EAV . Cellular and molecular differences between HFpEF and HFrEF: a step ahead in an improved pathological understanding. Cells. 2020;9:242. doi: 10.3390/cells9010242 31963679 PMC7016826

[85] Schiattarella GG , Rodolico D , Hill JA . Metabolic inflammation in heart failure with preserved ejection fraction. Cardiovasc Res. 2021;117:423–434. doi: 10.1093/cvr/cvaa217 32666082 PMC8599724

[86] Santhanakrishnan R , Chong JP , Ng TP , Ling LH , Sim D , Leong KT , Yeo PS , Ong HY , Jaufeerally F , Wong R , et al. Growth differentiation factor 15, ST2, high‐sensitivity troponin T, and N‐terminal pro brain natriuretic peptide in heart failure with preserved vs. reduced ejection fraction. Eur J Heart Fail. 2012;14:1338–1347. doi: 10.1093/eurjhf/hfs130 22869458

[87] Cheng JM , Akkerhuis KM , Battes LC , van Vark LC , Hillege HL , Paulus WJ , Boersma E , Kardys I . Biomarkers of heart failure with normal ejection fraction: a systematic review. Eur J Heart Fail. 2013;15:1350–1362. doi: 10.1093/eurjhf/hft106 23845797

[88] D'Elia E , Vaduganathan M , Gori M , Gavazzi A , Butler J , Senni M . Role of biomarkers in cardiac structure phenotyping in heart failure with preserved ejection fraction: critical appraisal and practical use. Eur J Heart Fail. 2015;17:1231–1239. doi: 10.1002/ejhf.430 26493383

[89] Sanders‐van Wijk S , van Empel V , Davarzani N , Maeder MT , Handschin R , Pfisterer ME , Brunner‐La Rocca HP . Circulating biomarkers of distinct pathophysiological pathways in heart failure with preserved vs. reduced left ventricular ejection fraction. Eur J Heart Fail. 2015;17:1006–1014. doi: 10.1002/ejhf.414 26472682

[90] Matsubara J , Sugiyama S , Nozaki T , Sugamura K , Konishi M , Ohba K , Matsuzawa Y , Akiyama E , Yamamoto E , Sakamoto K , et al. Pentraxin 3 is a new inflammatory marker correlated with left ventricular diastolic dysfunction and heart failure with normal ejection fraction. J Am Coll Cardiol. 2011;57:861–869. doi: 10.1016/j.jacc.2010.10.018 21310324

[91] Kresoja KP , Rommel KP , Wachter R , Henger S , Besler C , Klöting N , Schnelle M , Hoffmann A , Büttner P , Ceglarek U , et al. Proteomics to improve phenotyping in obese patients with heart failure with preserved ejection fraction. Eur J Heart Fail. 2021;23:1633–1644. doi: 10.1002/ejhf.2291 34231954

[92] Chia YC , Kieneker LM , van Hassel G , Binnenmars SH , Nolte IM , van Zanden JJ , van der Meer P , Navis G , Voors AA , Bakker SJL , et al. Interleukin 6 and development of heart failure with preserved ejection fraction in the general population. J Am Heart Assoc. 2021;10:e018549. doi: 10.1161/jaha.120.018549 33998283 PMC8483531

[93] Van Tassell BW , Trankle CR , Canada JM , Carbone S , Buckley L , Kadariya D , Del Buono MG , Billingsley H , Wohlford G , Viscusi M , et al. IL‐1 blockade in patients with heart failure with preserved ejection fraction. Circ Heart Fail. 2018;11:e005036. doi: 10.1161/circheartfailure.118.005036 30354558 PMC6545106

[94] Sanders‐van Wijk S , Tromp J , Beussink‐Nelson L , Hage C , Svedlund S , Saraste A , Swat SA , Sanchez C , Njoroge J , Tan RS , et al. Proteomic evaluation of the comorbidity‐inflammation paradigm in heart failure with preserved ejection fraction: results from the PROMIS‐HFpEF study. Circulation. 2020;142:2029–2044. doi: 10.1161/circulationaha.120.045810 33034202 PMC7686082

[95] Abegunde SO , Buckstein R , Wells RA , Rauh MJ . An inflammatory environment containing TNFα favors Tet2‐mutant clonal hematopoiesis. Exp Hematol. 2018;59:60–65. doi: 10.1016/j.exphem.2017.11.002 29195897

[96] Mann DL , McMurray JJ , Packer M , Swedberg K , Borer JS , Colucci WS , Djian J , Drexler H , Feldman A , Kober L , et al. Targeted anticytokine therapy in patients with chronic heart failure: results of the Randomized Etanercept Worldwide Evaluation (RENEWAL). Circulation. 2004;109:1594–1602. doi: 10.1161/01.CIR.0000124490.27666.B2 15023878

[97] Chung ES , Packer M , Lo KH , Fasanmade AA , Willerson JT , Anti TNFTACHFI . Randomized, double‐blind, placebo‐controlled, pilot trial of infliximab, a chimeric monoclonal antibody to tumor necrosis factor‐alpha, in patients with moderate‐to‐severe heart failure: results of the anti‐TNF Therapy Against Congestive Heart Failure (ATTACH) trial. Circulation. 2003;107:3133–3140. doi: 10.1161/01.CIR.0000077913.60364.D2 12796126

[98] Adamo L , Rocha‐Resende C , Prabhu SD , Mann DL . Reappraising the role of inflammation in heart failure. Nat Rev Cardiol. 2020;17:269–285. doi: 10.1038/s41569-019-0315-x 31969688

[99] Sano S , Horitani K , Ogawa H , Halvardson J , Chavkin NW , Wang Y , Sano M , Mattisson J , Hata A , Danielsson M , et al. Hematopoietic loss of Y chromosome leads to cardiac fibrosis and heart failure mortality. Science. 2022;377:292–297. doi: 10.1126/science.abn3100 35857592 PMC9437978

[100] Mas‐Peiro S , Abplanalp WT , Rasper T , Berkowitsch A , Leistner DM , Dimmeler S , Zeiher AM . Mosaic loss of Y chromosome in monocytes is associated with lower survival after transcatheter aortic valve replacement. Eur Heart J. 2023;44:1943–1952. doi: 10.1093/eurheartj/ehad093 36932691 PMC10232276

