# Supplementary material for: Clonal Hematopoiesis of Indeterminate Potential From a Heart Failure Specialist's Point of View
Source: J Am Heart Assoc. 2023 Jul 25;12(15):e030603. doi: 10.1161/JAHA.123.030603 (PMC10492961; doi:10.1161/JAHA.123.030603)
Supplement: Supplementary file 1 — Table S1 [file JAH3-12-e030603-s001.pdf]

Supplemental Material

Table S1. murine studies on the effect of clonal hematopoiesis in heart failure.

First author (year)	Cases	Controls	Additional interventions	Outcome
Sano [10] (2018)	Mice with bone marrow radiation followed by bone marrow transplantation with *Tet2* deficient (*Tet2* +/- or *Tet2* -/-) or *Dnmt3a* loss-of-function cells	Mice with bone marrow radiation followed by bone marrow transplantation with control cells	Angiotensin II in a subgroup of mice.	Bone marrow transplantation with *Tet2*-deficient or *Dnmt3a* loss-of-function mutated cells led to increased cardiac inflammation (IL-1, IL-6, CXCL1, CXCL2), hypertrophy and fibrosis and a decreased ejection fraction, compared to controls.
Sano [11] (2018)	Mice with bone marrow radiation followed by bone marrow transplantation with *Tet2* deficient (*Tet2* +/- or *Tet2* -/-) cells, additionally these mice TAC or ligation of the LAD.	Mice with bone marrow radiation followed by bone marrow transplantation with control cells, additionally these mice had TAC or ligation of LAD.	Inhibition of the NLRP3-inflammasome with MCC950 in a subgroup of mice.	Bone marrow transplantation with *Tet2*-deficient cells led to decreased ejection fraction and increased inflammation (IL-1, CXCL2, CD45) and fibrosis in mice with TAC or ligation of the LAD. The NLPR3-inflammasome inhibitor MCC950 reversed post-infarction and pressure overload remodeling caused by the CHIP-driver mutation in *Tet2*.

Table S1 (continued): murine studies on the effect of clonal hematopoiesis in heart failure

First author (year)	Cases	Controls	Additional interventions	Outcome
Sano [12] (2019)	Non-radiated mice with bone marrow transplantation from *Jak2* (V617F) mutated mice, additionally these mice had TAC or ligation of the LAD.	Non-irradiated mice with bone marrow transplantation from wild-type mice, additionally these mice had TAC or ligation of the LAD.	None	Bone marrow transplantation with *Jak2*(V617F) mutated bone marrow cells led to increase cardiac inflammation (IL-1, IL-6, TNF-alpha) and fibrosis and a decreased ejection fraction in mice with TAC or ligation of the LAD. *Jak2* (V617F) expressing HSPCs displayed a competitive advantage over *Jak2* (wild-type) HSPCs that was highly restricted to the myeloid lineage and shown as an increase of mutated neutrophils and monocytes in the blood.
Wang [14] (2020)	Non-radiated mice with bone marrow transplantation from *Tet2* deficient mice (*Tet2* +/- or *Tet2* -/-)	Non-irradiated mice with bone marrow transplantation from wild-type (*Tet2* +/+) mice	None	Bone marrow transplantation with *Tet2*-deficient cells led to progressive expansion of *Tet2*-deficient HSPCs as well as expansion of *Tet2*-deficient bone marrow derived myeloid cells within the heart, without an significant effect on yolk sac-derived cardiac-resident macrophages. Consecutively, there was a reduction in ejection fraction parallel to an increase in hypertrophy. Of note: this was the first study that showed a detrimental effect of the *Tet2*-driver mutation without an external injury (LAD ligation, TAC, or infusion with angiotensin II) or *Ldlr* knockout.

Table S1 (continued): murine studies on the effect of clonal hematopoiesis in heart failure

First author (year)	Cases	Controls	Additional interventions	Outcome
Yura [15] (2021)	Mice with bone marrow radiation followed by bone marrow transplantation with *ppm*1d gain-of-function mutation	Mice with bone marrow radiation followed by bone marrow transplantation with control cells	Angiotensin II and the NLRP3-inflammasome inhibitor MCC950 in a subgroup of mice.	The ppm1d mutation reduced left ventricle fraction shortening at 4 weeks and lead to an higher fibrotic cardiac tissue area parallel to higher bone marrow-derived (CCR2) macrophages/monocytes with higher IL1-β and IL-6 expression. This was abrogated by CCR2 knock-out or by the NLRP3 inflammasome inbibitor MCC950.
Sano [13] (2021)	Non-radiated mice with bone marrow transplantation from *Trp53* heterozygous-deficient (*Trp53*+/-) donor mice	Non-irradiated mice with bone marrow transplantation from *Trp53* homozygous wild type (*Trp53*+/+) donor mice	Doxorubicin in a subgroup of mice.	Doxorubicine led to expansion of *Trp53*-deficient HSPCs, myocardial neutrophil infiltration that produced IL-1β, IL-6 and TNF-α (without increased monocyte infiltration), and worse left ventricle systolic function. Neutrophil depletion prevented cardiac dysfunction.

CD = cluster of differentiation; CHIP = clonal haematopoiesis of indeterminate potential; CCR2 = C-C chemokine receptor 2; CXCL = chemokine (X-C-X) motif ligand; DNMT3A = DNA (cytosine-5)-methyltransferase 3A; HSPCs = hematopoietic stem and progenitor cells; IL = interleukin; JAK2 = Janus Kinase 2; LAD = left anterior descending artery; LDLR = low density lipoprotein receptor; NLRP3 = NLR family pyrin domain containing 3; TAC = transverse aortic constriction; TET2 = Tet methylcytosine dioxygenase 2; TNF = tumor necrosis factor; Trp53 = transformation related protein 53.
